# Exosomes derived from MSCs ameliorate retinal laser injury partially by inhibition of MCP-1

**DOI:** 10.1038/srep34562

**Published:** 2016-09-30

**Authors:** Bo Yu, Hui Shao, Chang Su, Yuanfeng Jiang, Xiteng Chen, Lingling Bai, Yan Zhang, Qiutang Li, Xiaomin Zhang, Xiaorong Li

**Affiliations:** 1 Tianjin Medical University Eye Hospital, Eye Institute & School of Optometry and Ophthalmology, Tianjin 300384, China; 2 Department of Ophthalmology and Visual Sciences, Kentucky Lions Eye Center, University of Louisville, Louisville, KY, USA

## Abstract

Although accumulated evidence supports the notion that mesenchymal stem cells (MSCs) act in a paracrine manner, the mechanisms are still not fully understood. Recently, MSC-derived exosomes (MSC-Exos), a type of microvesicle released from MSCs, were thought to carry functional proteins and RNAs to recipient cells and play therapeutic roles. In the present study, we intravitreally injected MSCs derived from either mouse adipose tissue or human umbilical cord, and their exosomes to observe and compare their functions in a mouse model of laser-induced retinal injury. We found that both MSCs and their exosomes reduced damage, inhibited apoptosis, and suppressed inflammatory responses to obtain better visual function to nearly the same extent *in vivo*. Obvious down-regulation of monocyte chemotactic protein (MCP)-1 in the retina was found after MSC-Exos injection. *In vitro*, MSC-Exos also down-regulated MCP-1 mRNA expression in primarily cultured retinal cells after thermal injury. It was further demonstrated that intravitreal injection of an MCP-1-neutralizing antibody promoted the recovery of retinal laser injury, whereas the therapeutic effect of exosomes was abolished when MSC-Exos and MCP-1 were administrated simultaneously. Collectively, these results suggest that MSC-Exos ameliorate laser-induced retinal injury partially through down-regulation of MCP-1.

Damage to retinal cells caused by injury, infection or ischemia triggers degeneration in neighboring nerve cells, resulting in the spread of morphological and functional retinal damage as well as irreversible visual impairment^1^. No effective neuroprotection therapy is currently available in the clinic for retinal injury. Research has shown that certain treatments are effective for the recovery of retinal cells. For example, stem cell transplantation^2^ and treatments with 7,8-dihydroxyflavone^3^, antagonist of *N*-methyl-d-aspartic acid receptors^4^, Ca2+ channel blockers^5^, anti-inflammatory agent^6^, signalling pathway inhibitors^7^ and neurotrophic factors^8^,^9^ show neuroprotective effects in various retinal disease models. However, none of these approaches have been successfully applied in the clinic.

MSCs are easily accessible primary cells possessing various biological functions such as multi-lineage differentiation, anti-inflammation, immune suppression, and neuroprotection^10^,^11^,^12^. MSCs have demonstrated particular promise for the treatment of retinal diseases such as retinal injury^13^, retinal degeneration^14^, and autoimmune uveitis^15^. It is accepted that MSCs function in a paracrine manner *in vivo* and not by direct differentiation^16^,^17^. In addition to soluble factors, exosomes are now believed to be important mediators for the paracrine effect of MSCs. Exosomes are 40–100-nm microvesicles with a bi-lipid membrane and cargo of abundant proteins and RNAs. They can be secreted by all cell types and are now recognized as natural vehicles involved in intercellular communication by protein and RNA delivery^18^. MSC-derived exosomes (MSC-Exos) possess similar functions to MSCs^19^. Despite the apparent advantages of MSC transplantation, the issues of allogeneic and xenogeneic immunological rejection, malignant transformation, and the risk of lodging and obstructing small vessels still exist^20^,^21^. All of these potential risks might be avoided by exosome administration^22^. Furthermore, exosomes, considering their nano-dimension, can easily pass through biological barriers and enter target organs^23^.

We previously demonstrated that intravenous injection of MSCs rescues retinal laser damage by inhibition of apoptosis and inflammatory responses^24^. In the present study, we compared the effect of intravitreal injection of MSC-Exos and MSCs using an animal model of laser-induced retinal injury. We found that the protective effect of MSC-Exos on injured retina was equal to that of MSCs in limiting the damage extent, reducing apoptosis, and inhibiting inflammatory responses. Retinal expression of monocyte chemotactic protein (MCP)-1 was significantly down-regulated by exosome therapy. Further experiments revealed that blocking MCP-1 ameliorated retinal injury, whereas injection of MCP-1 abolished the protective effect of MSC-Exos, suggesting that MSC-Exos reduce retinal impairment partially by targeting MCP-1.

## Results

### Isolation and characterization of exosomes

**Figure 1:**
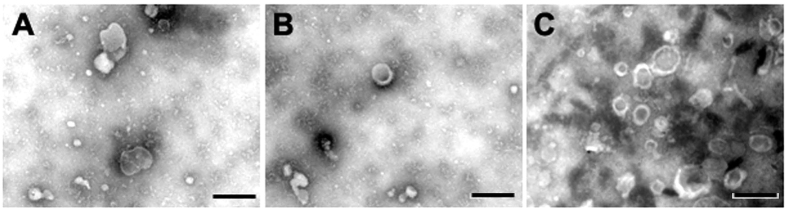
Micrographs of scanning electron microscopy of (**A**) maMSC-Exos, (**B**) hucMSC-Exos and (**C**) f-Exos show spheroid shaped vesicles at the diameter of about 40–100 nm. Scale bar = 200 nm.

MSCs isolated from mouse adipose tissue (maMSCs) were characterized by positivity for CD29, CD44, CD73, and Sca-1, and negativity for CD34, CD45, and CD11b. Human umbilical cord MSCs (hucMSCs) were positive for expression of CD13, CD44, CD73, and CD166, but negative for CD34 and CD45. Exosomes were isolated from the cells as described above, and observed under a scanning electron microscope. The spheroidal vesicles were 40–100 nm in diameter (Fig. 1). In addition, exosomal markers, such as CD9, CD63, and CD81, were detected by proteomic analysis.

### MSC-Exos diffuse rapidly and reach the retina after intravitreal injection

To examine the permeability and dynamic distribution of MSC-Exos in the retina, MSC-Exos were labeled with PKH26 before intravitreal injection. Frozen sections were prepared and DAPI staining was performed for observation by confocal microscopy. MSC-Exos started to be seen in the retina at 30 min after intravitreal injection. They diffused throughout the neural retina and retinal pigment epithelium (RPE) at 60 min and gradually spread to the outer layers. The control group showed no red fluorescence during the observations up to 120 min (see Supplementary Fig. S1).

### MaMSC-Exos alleviate retinal laser injury as efficiently as maMSCs

**Figure 2:**
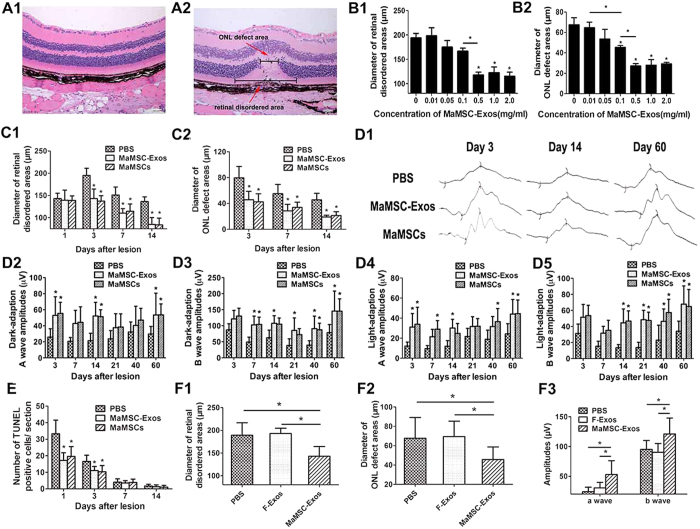
MaMSCs and maMSC-Exos promote the recovery of laser-induced retinal injury. (**A**) Micrographs of retinal histological images (H&E staining) before injury (A1) and 3 days after injury (A2). Main histological parameters are presented with lines. Scale bar = 50 μm. (**B**) Effect of maMSC-Exos at different concentrations on diameter of retinal disordered area (B1) and ONL defect area (B2) at 3^rd^ day post-injury. (**C**) Lesion areas after treatment with PBS, maMSCs or maMSC-Exos on 1^st^, 3^rd^, 7^th^ and 14^th^ days post-injury. n > 6, *p < 0.05. (D1) Amplitudes of dark-adapted ERG at 3, 14 and 60 days post-injury of PBS, maMSC- or maMSC-Exo-treated group. A wave and b wave amplitudes of dark adapted ERG (D2,D3) and light-adapted ERG (D4,D5) at 3, 7, 14, 21, 40 and 60 days post-injury were analysed and compared among the three groups. n = 7, *p < 0.05 (**E**) TUNEL staining of injury sections treated with PBS, maMSCs or maMSC-Exos at 1, 3, 7 and 14 days post-injury. n = 8, *p < 0.05. (**F**) Diameter of retinal disordered areas (F1), diameter of ONL defect areas (F2) and ERG responses (F3) at 3 days post-injury of maMSC-Exo- and f-Exo-treated groups. n = 6, *p < 0.05.

Our previous study showed that MSCs protect retinal cells from laser-induced damage^24^. Exosomes have been suggested to play an active role in mediating MSC functions^19^. These observations prompted us to consider the possibility that MSC-Exos might contribute to retinal protection. First, we examined the effects of various concentrations of maMSC-Exos on laser-induced retinal injury. Laser spots were generated with krypton laser on the right retina of each mouse, with diameter of 100 μm and intervals of more than 200 μm. Separate lesions could be identified clearly with intervals of normal retina on day 3 and 7 post-injury (see Supplementary Fig. S2). On day 3 post-injury, a cellular membrane, which consisted of dispersed pigment granules, proliferating RPE cells, macrophages, and pigment-laden cells, had developed in subretinal areas. In the center of the lesion, we observed disruption and total loss of nuclei in the outer nuclear layers (ONLs). The two previously described histological parameters were measured as shown in Fig. 2 A2. These results showed a significant reduction in the extent of injury by intravitreal injection of 5 μL maMSC-Exos at ≥ 0.5 mg/mL based on the histological analysis shown in Fig. 2B, indicating that maMSC-Exos alleviate retinal laser injury.

**Figure 3:**
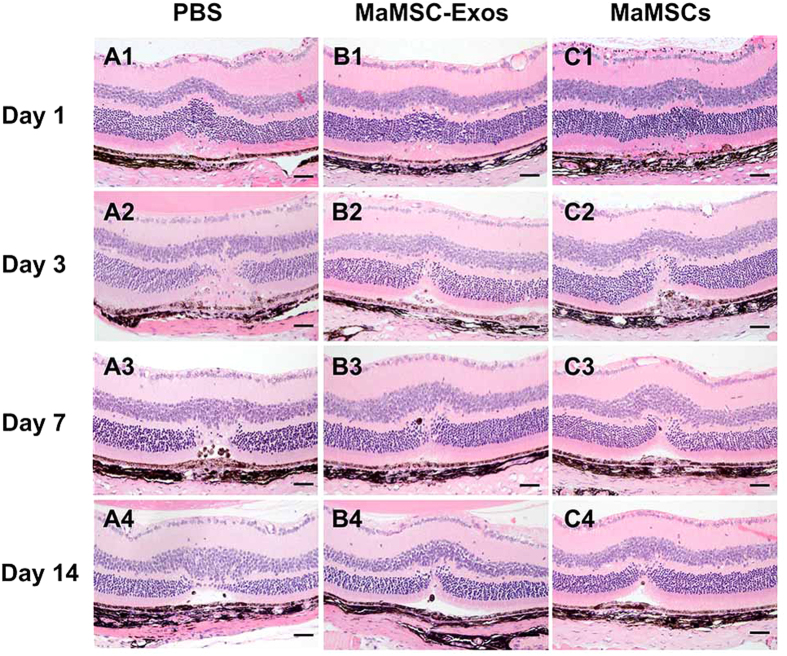
Representative examples of H&E staining images at different time points of evaluation. One day after injury of PBS (A1), maMSC-Exo (B1) or maMSC (C1) treated group. Three days after injury of PBS (A2), maMSC-Exo (B2) or maMSC (C2) treated group. Seven days after injury of PBS (A3), maMSC-Exo (B3) or maMSC (C3) treated group. Fourteen days after injury of PBS (A4), maMSC-Exo (B4) or maMSC (C4) treated group. Scale bar = 50 μm.

We next compared the effects of maMSC-Exos and maMSCs on laser-induced retinal injury to determine whether protection against injury by maMSCs can be achieved by maMSC-Exos. MaMSC-Exos at 0.5 mg/mL were applied in the following experiments. Images of hematoxylin and eosin (H&E) staining revealed morphological development of the lesion (Fig. 3). On day 1 post-injury, at the center of the lesion, ONLs showed paucity and disorder of their nuclei, but the inner retinal layers (INLs) showed no remarkable changes. Loss of pigment in the RPE was observed at the edges of the lesion, coinciding with its borders. Morphology changes on day 3 post-injury were the same as those described above. On day 7, the subretinal cellular membrane was still present. Cells in the INLs proliferated and migrated to the ONLs. On day 14, there were fewer pigment-laden cells that seemed to have moved internally from the subretinal space to ONLs, and a triangle-shaped subretinal space had formed. Except for day 1, maMSC- and maMSC-Exo-treated eyes showed equivalent amelioration with milder disorganization of the tissue, more residual photoreceptor cells, less inflammatory cell infiltration, smaller retinal disordered areas, and ONL defect areas compared with the control group (Fig. 2C), suggesting that the therapeutic efficacy of maMSC-Exos is equal with that of maMSCs.

In addition to the histology examination, we detected the improvement of dark- and light-adapted electroretinogram (ERG) responses in laser-injured mice treated with maMSCs or maMSC-Exos to evaluate retinal function. The dark- and light-adapted ERG responses had deteriorated post-injury (Fig. 2D1). Wave amplitudes of dark-adapted ERG responses on day 3, 14, and 60 in the treated groups were significantly larger than those in the control group. Except for the early stage of injury, wave amplitudes of light-adapted ERG responses and b-wave amplitudes of the treated groups were larger than those of the control group (Fig. 2D).

**Figure 4:**
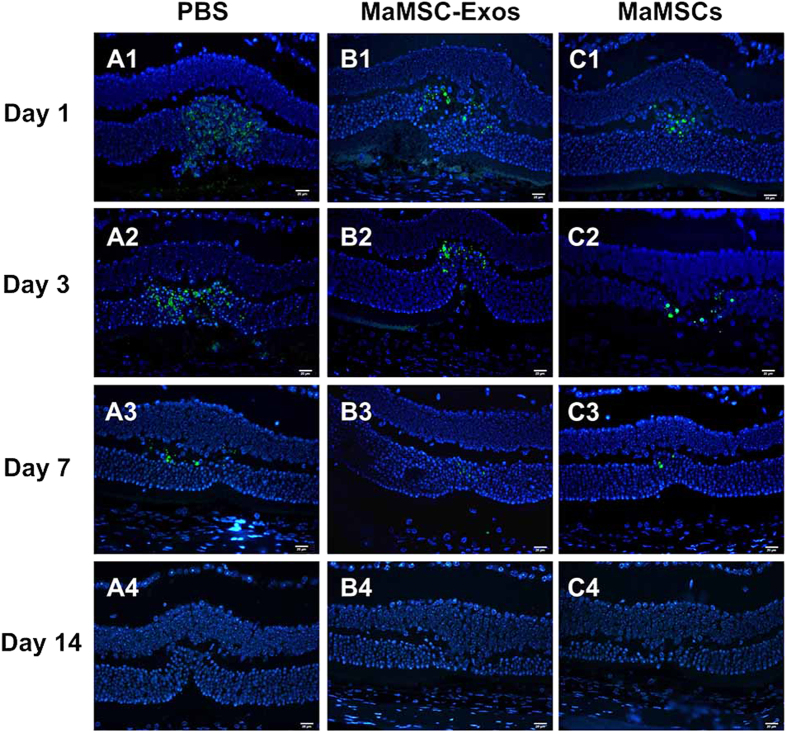
Representative examples of TUNEL staining images at different time points of evaluation. One day after injury of PBS(A1), maMSC-Exo (B1) or maMSC (C1) treated group. Three days after injury of PBS (A2), maMSC-Exo (B2) or maMSC (C2) treated group. Seven days after injury of PBS (A3), maMSC-Exo (B3) or maMSC (C3) treated group. Fourteen days after injury of PBS(A4), maMSC-Exo (B4) or maMSC (C4) treated group.(Scale bar = 20 μm; green: TUNEL staining of apoptotic cells; blue: DAPI staining of nucleus).

Apoptosis was examined by a terminal deoxynucleotidyl transferase-mediated dUTP nick end labeling (TUNEL) assay. On day 1, massive numbers of apoptotic cells were observed mainly in ONLs. On day 3 post-injury, fewer apoptotic cells were detected and mostly located at the edge of the lesions, whereas on day 7 and 14, few TUNEL-positive cells were detected (Fig. 4). Treatment with maMSCs or maMSC-Exos was found to significantly reduce the number of apoptotic cells compared with the control group on day 1 and 3. Consistent with both the histology and ERG examination, the apoptotic cell numbers of the two treated groups showed no significant difference (Fig. 2E).

To assess whether this protective activity of maMSC-Exos is a characteristic feature of MSCs, we compared maMSC-Exos with exosomes derived from fibroblasts (f-Exos) in terms of their effects on laser-induced retinal injury. We found that MSC-Exos, but not f-Exos, significantly reduced the injury and improved the ERG response, suggesting that the protective activity is specific to MSC-Exos (Fig. 2F).

Taken together, our results showed that maMSC-Exos protect retinal cells from laser damage, suggesting that the protection effect conferred by maMSCs is achieved at least partly by maMSC-Exos.

### HucMSC-Exos also have a retinal protection activity

**Figure 5:**
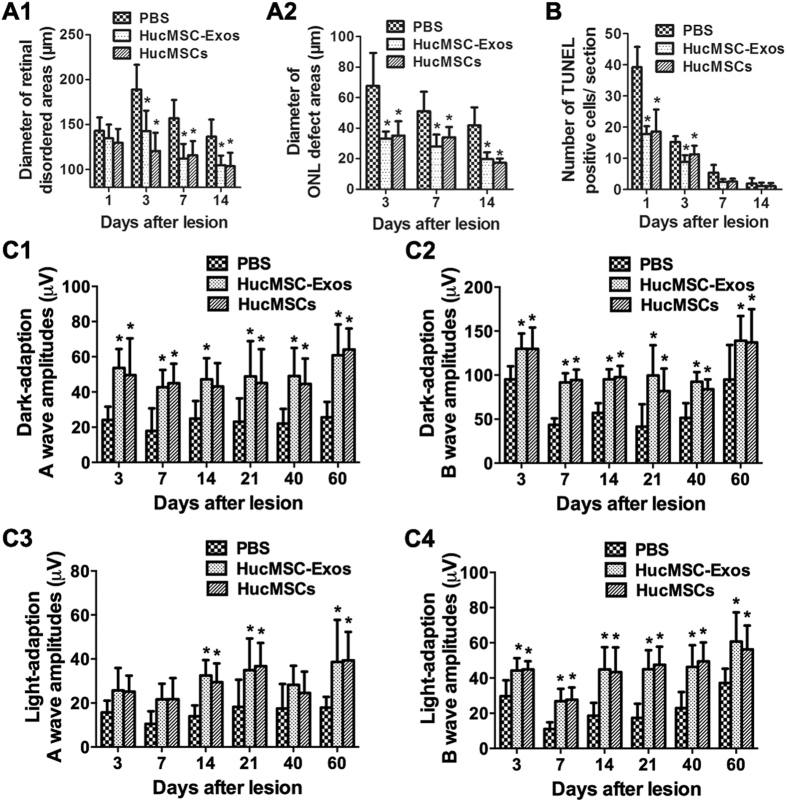
HucMSCs and hucMSC-Exos help protect retina in mouse retinal laser injury model. (**A**) Diameter of retinal disordered areas (A1) and ONL defect areas (A2) after treatment with PBS, hucMSCs or hucMSC-Exos at 1, 3, 7 and 14 days post-injury. n > 6, *p < 0.05. (**B**) Number of TUNEL positive cells/section of PBS-, hucMSC- and hucMSC-Exo-treated groups on 1^st^, 3^rd^, 7^th^ and 14^th^ days post-injury. n = 6, *p < 0.05. (**C**) A wave and b wave amplitudes of dark adapted ERG (C1,C2) and light-adapted ERG (C3,C5) at 3, 7, 14, 21, 40 and 60 days post-injury were analysed and compared among the three groups. n = 7, *p < 0.05.

Our results indicated that maMSC-Exos possess a retinal protection activity at a similar extent as that of maMSCs. MaMSC-Exos may provide an alternative therapeutic approach for wound healing, which might overcome the risks associated with stem cell therapy. Next, we determined whether hucMSC-Exos exhibit the same functions. Therefore, mice treated with hucMSCs or hucMSC-Exos were evaluated for histological and functional changes. Compared with those of the control group, retinas of the treated groups showed smaller lesioned areas (Fig. 5A), less TUNEL-positive cells (Fig. 5B), and better ERG responses (Fig. 5C), indicating that hucMSCs and hucMSC-Exos exert similar beneficial effects in the mouse retinal injury model.

### MSC-Exos suppress injury-induced inflammation

**Figure 6:**
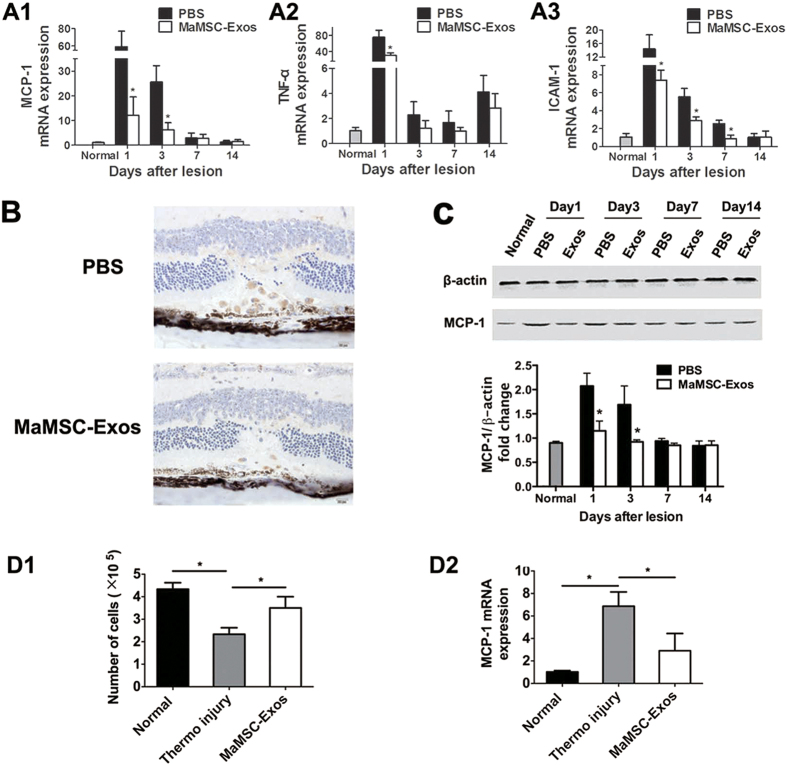
MaMSC-Exos suppress the injury-induced inflammation *in vivo* and *in vitro*. (A1–A3) Relative mRNA expression of MCP-1, TNF-α and ICAM1 of PBS- or MaMSC-Exo-treated group at 1, 3, 7 and 14 days post-injury. n = 7, *p < 0.05. (**B**) Immunohistochemistry staining images of PBS- or MaMSC-Exo-treated group at 3 days post-injury. (C1,C2) Western blotting analyses of MCP-1 in retina/RPE/Choroid tissues from PBS- or MaMSC-Exo-treated group at 1, 3, 7 and 14 days post-injury. n = 3, *p < 0.05. (D1) Cell number of normal, thermo injury or MaMSC-Exo-treated group at 3 days after thermo injury. (D2) Relative mRNA expression of MCP-1 in normal, thermo injury or MaMSC-Exo-treated group at 3 days after thermo injury.

Laser-induced acute inflammation contributes to tissue damage^26^. Therefore, we examined the relative mRNA expression of MCP-1, tumor necrosis factor-α (TNF-α), and intercellular adhesion molecule-1 (ICAM1) in retina/RPE/choroid tissues before and after laser injury by quantitative real-time PCR (qRT-PCR). The expression levels showed dynamic changes at various time points after injury. At the early phase, their gene expression increased significantly compared with the control group. MCP-1 and TNF-α mRNA levels were significantly down-regulated in the maMSC-Exo-treated group on day 1 and 3, and the ICAM1 mRNA level was decreased significantly on day 1, 3 and 7 compared with the control group (Fig. 6A). Among the tested inflammatory factors, expression of MCP-1 showed the most striking decrease after MSC-Exo therapy. Therefore, we confirmed the change of MCP-1 expression at the protein level. Consistent with the change in gene expression, on day 1 and 3, MCP-1 protein expression in the treated group was significantly lower than that in the control group (Fig. 6C).

**Figure 7:**
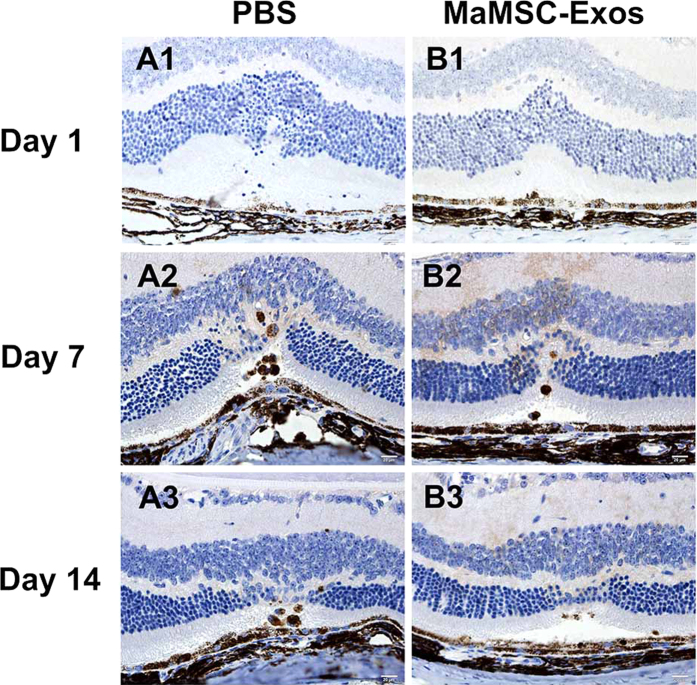
Representative examples of immunohistochemistry staining images at different time points of evaluation. One day after injury of PBS (A1) or maMSC-Exo (B1) treated group. Seven days after injury of PBS (A1) or maMSC-Exo (B1) treated group. Fourteen days after injury of PBS (A1) or maMSC-Exo (B1) treated group. Scale bar = 20 μm.

To further assess the inflammatory response to laser injury, we examined the distribution of macrophages, the major inflammatory cell type identified in retinal damage. Immunohistochemical staining for a monocyte/macrophage surface marker, CD68, was carried out to detect monocyte/macrophage infiltration after laser injury. CD68-positive cells mainly existed between RPEs and ONLs. Compared with the control group, fewer macrophages were detected in the MSC-Exo-treated group on day 3 (Fig. 6B), and few were detected on day 1, 7 and 14 (Fig. 7).

Our data showed that MCP-1 expression preceded and was coincident with maximal macrophage infiltration. Considering the central role of MCP-1 in recruitment of monocytes and photoreceptor degeneration^27^, we hypothesized that MSC-Exos regulate retinal impairment by regulating MCP-1. To test this hypothesis, we established a thermal injury model using primary cultured retinal cells to further validate the inhibitory effects of MSC-Exos on MCP-1 expression. The primary retinal cells were subjected to thermal injury in the presence or absence of maMSC-Exos. At 72 hours post-injury, the number of cells had decreased and the mRNA level of MCP-1was up-regulated. Indeed, exosomes remarkably reduced cell death and MCP-1 mRNA expression compared with the control group (Fig. 6D).

### MSC-Exos exert their therapeutic effect partially by inhibition of MCP-1 expression

**Figure 8:**
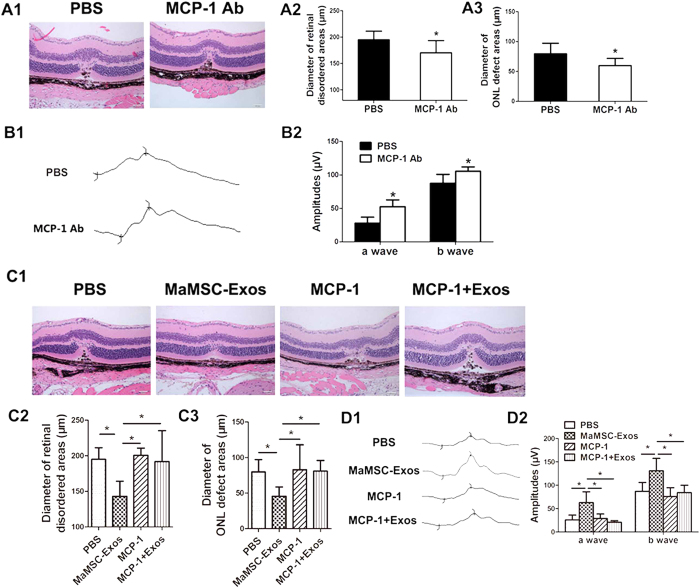
Effect of MCP-1 regulation on laser induced retinal injury in mice. (A1) Representative H&E staining images of PBS- or MCP-1 neutralizing antibody-treated groups at 3 days post-injury. Scale bar = 50 μm. Diameter of retinal disordered areas (A2) and diameter of ONL defect areas (A3) of PBS- or MCP-1 neutralizing antibody-treated group at 3 days post-injury. n = 6, *p < 0.05. (B1) Amplitudes of dark-adapted ERG at 3 days post-injury of PBS- or MCP-1 neutralizing antibody-treated group. (B2) Dark adapted ERG wave amplitudes of PBS- or MCP-1 neutralizing antibody-treated group at 3 days post-injury. n = 6, *p < 0.05. (C1) Representative H&E staining images of PBS-, maMSC-Exo-, MCP-1- or MCP-1+maMSC-Exo-treated group at 3 days post-injury. Scale bar = 50 μm. Diameter of retinal disordered areas (C2) and diameter of ONL defect areas (C3) of the four groups at 3 days post-injury. n = 6, *p < 0.05. (D1) Amplitudes of dark-adapted ERG at 3 days post-injury of PBS-, maMSC-Exo-, MCP-1- or MCP-1+maMSC-Exo-treated group. (D2) Dark adapted ERG wave amplitudes of PBS-, MaMSC-Exo-, MCP-1- or MCP-1+MaMSC-Exo-treated group at 3 days post-injury. n = 6, *p < 0.05.

The change in MCP-1 expression of MSC-Exo-treated eyes raised the interesting possibility that it plays a partial role in the retinal injury response. Therefore, we determined whether the change in MCP-1 expression affected laser-induced retina injury. First, we characterized and compared the retinal injury in MCP-1-Ab- and PBS-treated mice to investigate whether MCP-1 down-regulation alleviated retinal laser injury. The agents were administered intravitreally immediately after injury. We consistently observed smaller diameters of retinal disordered and ONL defect areas in the treated group on day 3 (Fig. 8A), as well as elevations of a and b wave amplitudes of dark-adapted ERG responses (Fig. 8B) compared with the control group. We concluded that MCP-1 inhibition protects retinal cells from laser-induced damage.

In contrast, we increased the local concentration of MCP-1 by intravitreal injection of MCP-1 with or without MSC-Exos. Although an increase in MCP-1 did not exacerbate injury further, it significantly abolished the MSC-Exo treatment effect. Among the four groups, only the MSC-Exo-treated group showed beneficial effects such as milder histological changes and higher amplitudes of ERG waves, indicating that the protective effect of MSC-Exos on retinal laser injury was abolished by up-regulation of MCP-1 (Fig. 8C,D).

Taken together, our results demonstrate that suppression of MCP-1 induction during laser induced retina injury reduces retina tissue damage, suggesting that MSC-Exos may afford the tissue protection activity partially through MCP-1 inhibition.

## Discussion

MSC therapy is currently being translated into clinical application to a variety of diseases, and the mechanisms underlying their therapeutic effects are mainly attributed to a paracrine pattern. However, no one factor has been proven to be sufficient to mediate their effects. It has been found that complexes with a diameter of 50–100 nm in MSC-conditioned medium are factors that mediate cardio-protective effects in pig and mouse myocardial ischemia/reperfusion injury models^16^. After they were demonstrated to be exosomes^28^, these complexes were widely tested for the treatment of various diseases such as acute kidney injury^29^, graft-versus-host disease^30^, skin damage^31^, and stroke^32^. In myocardial ischemia/reperfusion injury, they were found to reduce the infarct size and oxidative stress. In skin damage, exosomes accelerate re-epithelialization, promote proliferation, and inhibit apoptosis of skin cells *in vivo* and *in vitro*. It has also been shown that intravenous administration of MSC-Exos post-stroke enhances neurite remodeling and neurogenesis, and improves functional recovery^22^. Collectively, MSC-Exos are being increasingly recognized as appealing candidates to repair tissue damage and protect neurocytes.

Laser-induced retinal injury model is widely applied in retinal damage study. In the current study, the wavelength, power and exposure time of the laser were precisely controlled to avoid breaking Bruch’s membrane. Simillar laser lesions (spot size 200 μm) had been produced in rats in other studies^33^,^34^. In our experiments, twenty laser spots were generated on each retina in such a manner that every laser spot is at least two-spot diameters (200 μm) away from others. The relatively large numbers of laser spots in our study not only make it easier to obtain enough histological data from equal number of samples, but also make it possible to detect changes of retinal function by ERG evaluation. In another paper to determine the functional changes after laser photocoagulation using rats, one hundred and twenty-nine laser spots (spot size 200 μm) covering a quarter of the retina per rat eye were applied, and separate lesions could be identified microscopically at each of the three time points (3, 20, and 60 days after exposure to laser)^35^. In our study, separate lesions spaced by normal retina could also be identified clearly under microscope with 20 laser spots generated in one mouse eye, indicating that this model of mice is suit for the study of retinal injury.

In the current study, we successfully isolated exosomes from fibroblasts and MSCs of different origins with widely applied methods^36^. Our data suggested that, in a mouse model of retinal laser injury, intravitreal injection of MSC-Exos had a comparable therapeutic effect as MSCs in limiting damage progression, reducing cell apoptosis, and improving visual function. *In vitro* experiments indicated that MSC-Exos reduced retinal cell loss induced by heat injury. These results further support the notion that the retinal protective effects of MSCs are mediated by a paracrine manner, rather than direct trans-differentiation and repopulation. Studies have demonstrated that laser injury ablates the RPE and causes photoreceptor damage, leading to the responsive release of pro-inflammatory mediators, such as MCP-1, TNF-α, and ICAM1, and the migration and infiltration of inflammatory cells^37^,^38^. We found increases in the mRNA expression of these factors at the early stage after injury, which was strongly suppressed by both MSCs and MSC-Exos, suggesting that MSCs played an important role in suppression of inflammation by exosomes. Among these factors, MCP-1 mRNA expression showed the most striking decrease in the treated groups.

MCP-1 is currently one of the most extensively studied chemotactic cytokines, which was primarily found to be functional in regulating migration and infiltration of monocytes/macrophages^39^. It can be produced by a variety of cell types, especially monocytes/macrophages^40^. Retinopathies, such as retinal detachment^41^, retinal degeneration^42^, diabetic retinopathy^43^, and uveoretinitis^44^, induce MCP-1 secretion that activates and attracts local macrophages and microglial cells, leading to further tissue damage^27^. The activation of macrophages and microglia in turn promotes MCP-1 secretion. This series of reactions play an important role in the progression of retinal damage^45^,^46^, while MCP-1 inhibition or down-regulation had a beneficial effect on monocyte/macrophage-associated photoreceptor degeneration^47^,^48^. In our study, consistent with the expression of MCP-1, immunohistochemical staining for CD68, a monocyte/macrophage surface marker, revealed that macrophages aggregated at the damaged site after laser injury, whereas MSC-Exo therapy significantly reduced macrophage infiltration. Administration of MCP-1 alone did not aggravate the damage. However, when MCP-1 was injected together with MSC-Exos, MCP-1 largely abolished their therapeutic effects. Furthermore, an MCP-1-neutralizing antibody also provided therapeutic benefits against retinal injury. *In vitro* experiments further confirmed that MSC-Exos reduced MCP-1 expression and heat-induced apoptosis or death of retinal cells. These results support the important role of MCP-1 in the pathogenesis of retinal damage, and suggest that MSC-Exos exert their protective effect, at least partially, through regulation of MCP-1 and macrophage infiltration.

Exosomes can deliver a large cargo of proteins and RNAs to modify cell and organ functions^49^. Several studies have confirmed that miRNAs encapsulated in MSC-Exos are functional molecules that mediate beneficial effects^50^,^51^, while another study confirmed that the therapeutic effect was achieved by proteins^31^.

As bi-lipid membrane vesicles, exosomes protect the contents from degradative enzymes or chemicals. They facilitate the uptake of therapeutic proteins or RNAs into target cells through endocytosis or membrane fusion, regardless of biological barriers^52^. Intravitreal injection is a common route to treat retinal diseases, because it avoids the dilution effect and possible side effects to other organs associated with systemic delivery. MSC-Exos appear to be superior candidates for intravitreal injection, which might overcome risks and obstacles associated with stem cell transplantation therapy such as possible long-term pathological differentiation, vitreous opacity, and the inconvenience of preservation.

In summary, we have demonstrated that MSCs and MSC-Exos exert similar significant beneficial effects on retinal laser injury repair. MCP-1 down-regulation, which might be influenced by proteins or RNAs encapsuled in MSC-Exos, are important in this process. Our findings provide a possible therapeutic tool to protect the retina, as well as insights into the mechanism of MSCs in damage repair and neural protection. In light of our results, further studies are needed to better understand the direct relationship between the constituents of MSC-Exos and MCP-1 regulation.

## Methods

The study was approved by the Ethics Committee of Tianjin Medical University [2013KY(L)-09]. All experiments were carried out in accordance with the approved guidelines. Informed consent was obtained from all umbilical cord donors.

### Animals

Female (age range: 6–8 weeks), male (age range: 8–10 weeks), and newborn C57BL/6 mice were purchased from Peking University Health Science Center. The care and use of all animals in this study conformed to the regulations in the ARVO Statement for the Use of Animals in Ophthalmic and Vision Research, and were approved by the Laboratory Animal Care and Use Committee of the Tianjin Medical University. Adult animals were housed in a temperature and humidity-conditioned room under 12-hour light/12-hour dark cycles with free access to food and water.

### Cell culture

HucMSCs -After washing with PBS and cutting into small pieces, human umbilical cords were digested with collagenase II for 1 hour, followed by trypsinization for 20 minutes at 37 °C. Dissociated cells were collected and cultured in Dulbecco’s modified Eagle’s medium (DMEM)/F12 containing 10% fetal bovine serum, 100 U/ml penicillin, and 100 μg/ml streptomycin at 37 °C in humidified atmosphere with 5% CO_2_.

Mouse adipose MSCs -Male C57BL/6 mice were sacrificed to collect adipose tissue. After washing in α-minimum essential medium (αMEM) three times, the adipose tissue samples were cut into small pieces and digested with 0.1% collagenase II for 2 hours. Dissociated cells were collected and cultured in αMEM containing 20% fetal bovine serum, 100 U/ml penicillin, 100 μg/ml streptomycin, and MEM non-essential amino acids (Gibco, USA) at 37 °C in a humidified atmosphere with 5% CO_2_. MSCs at passage 3–5 were used to collect exosomes.

MaMSCs were tested for CD11b, CD29, CD34, CD44, CD45, CD73, and Sca-1 expression, and hucMSCs were tested for CD13, CD34, CD44, CD45, CD73, and CD166 expression by flow cytometry.

Fibroblasts were purchased from Shanghai Boye Biological Company. The cells were cultured in high glucose DMEM (Gibco) containing 10% fetal bovine serum, 100 U/ml penicillin, and 100 μg/ml streptomycin (Gibco) at 37 °C in a humidified atmosphere with 5% CO_2_.

Mouse retinal cells - Retinas from newborn C57BL/6 mice were collected and digested with 0.125% trypsin for 25 minutes at 37 °C. After collection by centrifugation at 1500 rpm for 5 minutes, the cells were cultured in DMEM/F12 containing 10% fetal bovine serum, 100 U/ml penicillin, and 100 μg/ml streptomycin at 37 °C in a humidified atmosphere with 5% CO_2_.

### Isolation and characterization of exosomes

Supernatants of fibroblasts or MSCs at passage 3–5 cultured in FBS-free medium were centrifuged at 200, 2000, and 10,000× *g* sequentially to remove cells and cell debris, then centrifuged at 110,000× *g* for 2 hours. After washing twice, the pellet was resuspended in PBS. The protein concentration of exosomes was measured by a BCA protein assay kit (Biorega, China). Scanning electron microscopy was used to observe the morphology of exosomes. Proteomic analysis was performed to identify surface markers.

### Retinal laser injury model and treatment

Mice were anesthetized with chloral hydrate at 500 mg/kg body weight intraperitoneally after pupil dilation with 0.5% tropicamide. A krypton laser (NOVUS OMNI, Coherent, USA; wavelength, 647 nm; power, 100 mw; exposure time, 100 ms; diameter, 100 μm) was used to generate 20 laser spots to the right retina of each mouse using a hand-held coverslip as the contact lens. Every spot was more than 200 μm away from others while avoiding major vessels. For treatment, 5 μL PBS alone, 1 × 10^4^ MSCs, MSC-Exos at various concentrations, 0.5 mg/mL exosomes derived from fibroblasts (F-Exos), 0.2 mg/mL MCP-1-neutralizing antibody (R&D, USA), 0.5 mg/mL MCP-1 (R&D), or PBS containing both MCP-1 and MSC-Exos were intravitreally injected immediately after laser injury. The left eyes were regarded as normal controls.

### Labeling and tracing of MSC-Exos

HucMSC-Exos were labeled with the red fluorescent dye PKH26 (Sigma–Aldrich, USA) according to the manufacturer’s instructions. Briefly, exosomes were resuspended in 1 mL Diluent C mixed with 4 *μ*L PKH26 and then incubated for 4 min at room temperature. An equal volume of exosome-free FBS was added to stop the reaction. After washing and resuspending in PBS, labeled exosomes or PBS alone were injected intravitreally immediately after laser injury. Frozen sections were prepared and DAPI staining was performed at 15, 30, 60 and 120 min after injection to determine the dynamic distribution of exosomes under a confocal microscope (Nikon, Japan).

### Histological analysis

The eyes were fixed, dehydrated and embedded in paraffin. H&E staining and immunohistochemistry were performed on 4-μm-thick serial coronal sections of the retinas. The sections at the centers of laser spots were selected from the successive sections. After antigen retrieval, non-specific binding was blocked by incubation in 5% bovine serum albumin. The sections were incubated with an antibody against mouse CD68 according to the (Abcam, UK) manufacturer’s instructions. Color development was performed with diaminobenzidine substrate (Zhong Shan Jin Qiao, China) following the manufacturer’s instructions. Observations were performed by light microscopy. For H&E staining, image processing software (CellSens Standard1.6) was used to measure the diameter of disordered retinal areas and ONL defects. The disordered retina area was defined as the area in which cytoarchitectural changes in any retinal layer could be distinguished, according to the method described previously^25^.

### TUNEL staining

Paraffin-embedded sections were deparaffinized. Apoptotic retinal cells were detected by an *in situ* cell death detection kit (Roche, Germany) according to the manufacturer’s protocol. All sections were counterstained with DAPI, followed by observation and counting under a fluorescence microscope.

### ERG

After dark adaption overnight, mice were anesthetized and then their pupils were dilated as described above. Carboxymethyl cellulose sodium eye drops and oxybuprocaine hydrochloride eye drops were administered afterwards. Two round electrodes were placed in direct contact with the cornea. Two needle electrodes placed subcutaneously on the forehead served as reference electrodes, and a subcutaneous needle electrode on the back was used as a ground electrode. The parameters of the photic stimulator of the dark-adapted ERG were set as follows: 2.92-millisecond flash time, 10-second intervals, and 3.0-cd*s/m^2^ flash intensity. Before light-adapted ERG detection, mice were light adapted in 30-cd/m^2^ background light for 5 minutes. The parameters of the stimulator were the same.

### qRT-PCR

Cornea, sclera, and lens tissues were dissected away from the enucleated eye. Total RNA was isolated from the remaining tissues (i.e. retina, RPE, and choroid) by Trizol reagent (Invitrogen, USA). One microgram of cDNA was synthesized from DNase-treated RNA using a revertaid first strand cDNA synthesis kit (Thermo, USA) according to the manufacturer’s protocol. qRT-PCR were carried out in 384-well plates in a total volume of 8 μL containing SYBR Green Master (Roche, Switzerland), reverse primers, and cDNA. Threshold cycles were normalized to expression of the housekeeping gene glyceraldehyde-3-phosphate dehydrogenase (GAPDH). The fold change was analyzed by the ^ΔΔ^Ct method. The primers were as follows: GAPDH: forward, 5′-TGTGTCCGTCGTGGATCTGA-3′, and reverse, 5′-CCTGCTTCACCACCTTCTTGA-3′; MCP-1: forward, 5′-CAGGTCCCTGTCATGCTTCTG-3′, and reverse, 5′-GAGCCAACACGTGGATGCT-3′; TNF-α: forward, 5′-GGTCCCCAAAGGGATGAGAA-3′, and reverse, 5′-TGAGGGTCTGGGCCATAGAA-3′; ICAM1: forward, 5′-CCCCGCAGGTCCAATTC-3′, and reverse, 5′-CAGAGCGGCAGAGCAAAAG-3′.

### Western blotting

Proteins were extracted from Retina/RPE/Choroid tissues. The protein concentration was determined using the BCA protein assay kit. Equal amounts of proteins were resolved by SDS-polyacrylamide gel electrophoresis using 12% denaturing gels and then transferred onto polyvinylidene fluoride membranes (Millipore, USA). The blotted membranes were incubated with a rabbit anti-CCL2 antibody (1:2000; Abcam, UK) or rabbit anti-β-actin antibody (1:200; Boshide, China) at 4 °C overnight. The membranes were washed with Tris-buffered saline/Tween (TBS/T) three times and then incubated with a peroxidase-conjugated goat anti-rabbit secondary antibody (1:5000; Zhong Shan Jin Qiao, China) at room temperature for 2 hours. The membranes were washed again with TBS/T three times. Signals were developed by chemiluminescence using an ECL kit (GE, USA). The blots were visualized by the BioSpectrum Imaging System (UVP, USA) and analyzed by Gene Tool software (Syngene, UK).

### Thermal injury of retinal cells

To subject the retinal cells with thermal injury, the culture medium was replaced with PBS that had been pre-warmed at 45 °C for 10 minutes and then changed back to normal medium at 37 °C in the presence or absence of 10 μg exosomes. After 3 days, the cell number and MCP-1 mRNA level were quantified.

### Statistical analysis

All experiments were repeated at least three times. Statistical analysis was performed using the Student’s *t*-test for two sets of data, one-way analysis of variance (ANOVA) or two-way ANOVA for three means. For each analysis, p < 0.05 was considered significant.

## Additional Information

**How to cite this article**: Yu, B. *et al*. Exosomes derived from MSCs ameliorate retinal laser injury partially by inhibition of MCP-1. *Sci. Rep.* **6**, 34562; doi: 10.1038/srep34562 (2016).

## Supplementary Material

Supplementary Information
