# Supplementary material for: Exosomes derived from MSCs ameliorate retinal laser injury partially by inhibition of MCP-1
Source: Sci Rep. 2016 Sep 30;6:34562. doi: 10.1038/srep34562 (PMC5043341; doi:10.1038/srep34562)
Supplement: Supplementary Information [file srep34562-s1.pdf]

Exosomes derived from MSCs ameliorate retinal laser injury partially by inhibition of MCP-1

Bo Yu[1], Hui Shao[2], Chang Su[1], Yuanfeng Jiang[1], Xiteng Chen[1], Lingling Bai[1], Yan Zhang[1], Qiutang Li[2], Xiaomin Zhang[*1], Xiaorong Li[*1]

[1]Tianjin Medical University Eye Hospital, Eye Institute & School of Optometry and Ophthalmology, Tianjin 300384, China
[2]Department of Ophthalmology and Visual Sciences, Kentucky Lions Eye Center, University of Louisville, Louisville, KY, USA

Supplementary figure S1

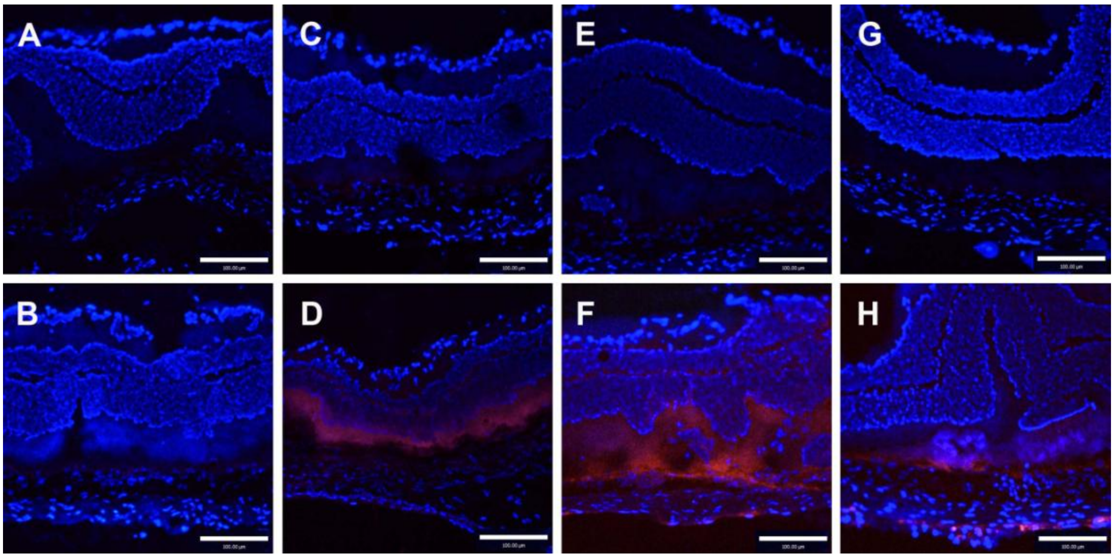

Supplementary figure S1. Huc-MSC-Exos diffused rapidly after intravitreal injection. Confocal microscopy image of frozen sections of PBS-treated eyes at 15min (A), 30 min (C), 60 min (E), and 120 min (G) after intravitreal injection. Confocal microscopy image of frozen sections of PKH26-labeled exosome-treated eyes at 15min (B), 30 min (D), 60 min (F), and 120 min (H) after intravitreal injection. (Scale bar =100μm; red: PKH26-labeled exosomes; blue: DAPI staining of nuclei)

Supplementary figure S2

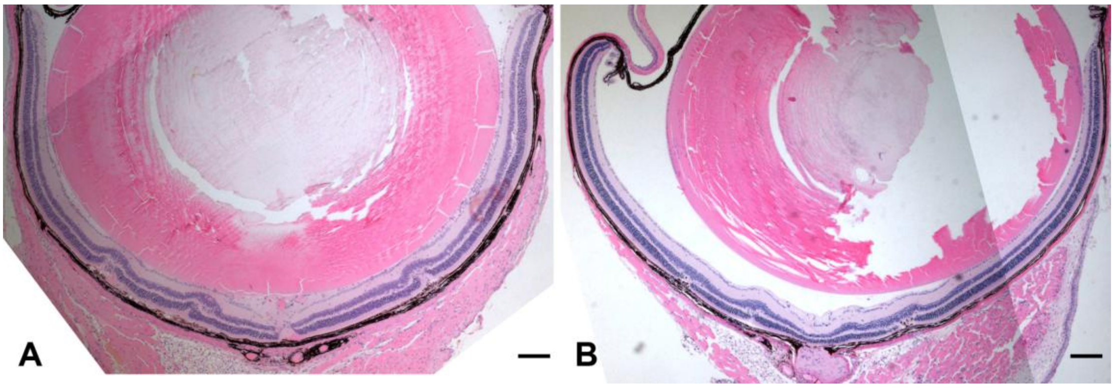

Supplementary figure S2. Typical pictures of the eye section after laser injury. (A) Eye section on day 3 post-injury; (B) Eye section on day 7 post-injury. Scale bar =200μm.
